# The iBerry study: a longitudinal cohort study of adolescents at high risk of psychopathology

**DOI:** 10.1007/s10654-021-00740-w

**Published:** 2021-04-01

**Authors:** Nina H. Grootendorst-van Mil, Diandra C. Bouter, Witte J. G. Hoogendijk, Stefanie F. L. M. van Jaarsveld, Henning Tiemeier, Cornelis L. Mulder, Sabine J. Roza

**Affiliations:** 1 Department of Psychiatry, Erasmus MC University Medical Center, Rotterdam, The Netherlands; 2 Epidemiological and Social Psychiatric Research Institute (ESPRi), Department of Psychiatry, Erasmus MC University Medical Center, Rotterdam, The Netherlands; 3 Department of Epidemiology, Erasmus MC University Medical Center, Rotterdam, The Netherlands; 4 Department of Child and Adolescent Psychiatry and Psychology, Erasmus MC, University Medical Center, Rotterdam, The Netherlands; 5 Department of Social and Behavioral Sciences, Harvard T.H. Chan School of Public Health, Boston, USA

**Keywords:** Epidemiology, Psychopathology, Cohort study, Design, Adolescents, High-risk

## Abstract

The iBerry study is a population-based cohort study designed to investigate the transition from subclinical symptoms to a psychiatric disorder. Adolescents were selected based on their self-reported emotional and/or behavioral problems assessed by completing the strengths and difficulties questionnaire-youth (SDQ-Y) in their first year of high school. A total of 16,736 SDQ-Y questionnaires completed in the academic years 2014–2015 and 2015–2016 by students in the greater Rotterdam area in the Netherlands were screened. A high-risk group of adolescents was then selected based on the 15% highest-scoring adolescents, and a low-risk group was randomly selected from the 85% lowest-scoring adolescents, with a 2.5:1 ratio between the number of high-risk and low-risk adolescents. These adolescents were invited to come with one parent for a baseline visit consisting of interviews, questionnaires, neuropsychological tests, and biological measurements to assess determinants of psychopathology. A total of 1022 high-risk and low-risk adolescents (mean age at the first visit: 15.0 years) enrolled in the study. The goal of the iBerry study is to follow these adolescents for a 10-year period in order to monitor any changes in their symptoms. Here, we present the study design, response rate, inclusion criteria, and the characteristics of the cohort; in addition, we discuss possible selection effects. We report that the oversampling procedure was successful at selecting a cohort of adolescents with a high rate of psychiatric problems based on comprehensive multi-informant measurements. The future results obtained from the iBerry Study will provide new insights into the way in which the mental health of high-risk adolescents changes as they transition to adulthood. These findings will therefore facilitate the development of strategies designed to optimize mental healthcare and prevent psychopathology.

**Supplementary Information:**

The online version contains supplementary material available at 10.1007/s10654-021-00740-w.

## Introduction

Psychiatric disorders are highly burdensome due to their prevalence, comorbidity, chronicity, and costs [1–6]. Psychiatric disorders are often preceded by non-specific symptoms in adolescence such as insomnia, difficulty with motivation, anergia, anxiety, and/or affective dysregulation [7, 8]. Mental health during adolescence shapes one’s later life with respect to all major domains, including health, social relationships, education, and employment. Although these symptoms can be transient and are often part of normal adolescent development, in some cases they constitute a prodromal phase or the onset of severe mental disorders [9]. Therefore, developing preventive and/or early intervention strategies requires a more thorough understanding of the pathways and processes that underlie the transition from these non-specific symptoms in adolescence to the development of a full-blown disorder in adulthood [10].

The etiology of a given disorder can be studied in participants from the general population, in an at-risk population, or in patients in the early stages of the disease. Studying the transition from subclinical symptomatology to a psychiatric disorder in the general population is often difficult because of the high rate of selective dropout among these participants [11]. Patient-based studies include patients who are already engaged in treatment and may therefore introduce selection bias (i.e., referral bias). In addition, a delay in treatment ranging from years to even decades after the onset of mental illness is relatively common [12], and up to two-thirds of adolescents who experience severe symptoms do not seek treatment [13, 14]. Selecting participants based on familial loading of psychopathology has proven effective with respect to identifying individuals who are at risk of developing a severe mental disorder such as a mood or psychotic disorder [15, 16]; however, because this approach does not identify individuals who develop a sporadic or non-familial form of mental illness, it selects for a particular inheritance pattern. Most previous studies focused on a specific psychiatric diagnosis, whereas other studies showed that the familial transmission of risk is only partly diagnosis-specific [15]; thus, many individuals who are at risk of developing a psychiatric disorder may not necessarily be represented in either population-based or patient-based studies.

Longitudinal cohort studies conducted over the past two decades have provided key insights into the epidemiology, risk factors, and trajectories of mental disorders [17–19]. In 2015, the iBerry (Investigating Behavioral and Emotional Risk in Rotterdam Youth) Study was initiated in order to investigate the etiology and course of psychopathology using a cross-diagnostic design. The aim of the iBerry Study is to investigate the bio-psychological development and determinants of psychiatric disorders in a contemporary population of adolescents. For this population-based cohort, adolescents were enrolled from the general population. By assessing their self-reported emotional and behavioral problems in their first year of high school, we oversampled adolescents with emotional and/or behavioral symptoms. Using this strategy, the incidence of psychiatric symptoms in the cohort will be increased, thereby increasing our ability to identify the developmental trajectories and causal pathways that underlie mental disorders.

## Study design

### General overview

The iBerry Study is a prospective longitudinal cohort study of adolescents at risk of developing psychopathology, conducted in the greater Rotterdam area of the Netherlands. This region includes an urban area (the city of Rotterdam), a suburban area, and relatively rural areas in the Netherlands. By screening a self-report questionnaire, adolescents with high emotional and behavioral problem scores were oversampled in the cohort. Our goal is to follow these adolescents in adulthood, inviting them for follow-up visits every 2–3 years.

### Eligibility and enrollment

In the Netherlands, all students in primary and secondary school receive general medical examinations as part of a standard preventive healthcare approach performed by community Child and Family Centers. The data used for this specific study were obtained from questionnaires administered to first-year students (ranging in age from 12 to 15 years) at secondary schools covered by the Child and Family Center of Rijnmond (CJG Rijnmond). In two consecutive academic years (2014–2015 and 2015–2016), parents and adolescents were informed in writing regarding the iBerry Study. Using a passive informed consent procedure, adolescents over the age of 12 years and their parents were informed that the strengths and difficulties questionnaire-youth (SDQ-Y) questionnaire would be used for research purposed unless they objected. Adolescents completed the SDQ-Y in a classroom setting under the supervision of a teacher and a nurse from the Child and Family Center; the nurse informed the adolescents that their answers would be kept strictly confidential. Adolescents with a score indicating a possible health risk were followed up during a subsequent physical examination administered by a nurse or a physician from the Child and Family Center. The typical annual response rate for this questionnaire is approximately 90% with illness-related absence of the student on the day the questionnaire was administered as the most common reason for non-response [16].

### Screening strategy

The SDQ-Y is one of the most widely used instruments for screening mental health in children and adolescents [20]. The SDQ-Y consists of 25 items scored on a three-point Likert scale, divided over the following five subscales: emotional symptoms, conduct problems, hyperactivity/inattention, peer problems, and prosocial behavior. Subscale scores were computed by summing the items in each subscale. To correct for a maximum of two missing items, the subscale score was multiplied by the number of items per subscale and divided by the number of scored items [21]. A total score was then calculated by adding the scores obtained for the emotional, conduct, hyperactivity/inattention, and peer problems subscales, with a total score ranging from 0 to 40 [22]. This total score provides a measure of overall mental health problems and has been shown to have good psychometric properties [23]. A high-risk group was then selected based on the highest-scoring adolescents in the top 15th percentile, and a low-risk group was randomly selected from the 85% lowest-scoring adolescents. To take into account potential gender differences in SDQ-Y scores, the percentiles for boys and girls were computed separately [21]. In total, our aim was to include 1,000 adolescents, oversampling the high-risk group at a ratio of 2.5:1.

### Screening and response rate

**Fig. 1:**
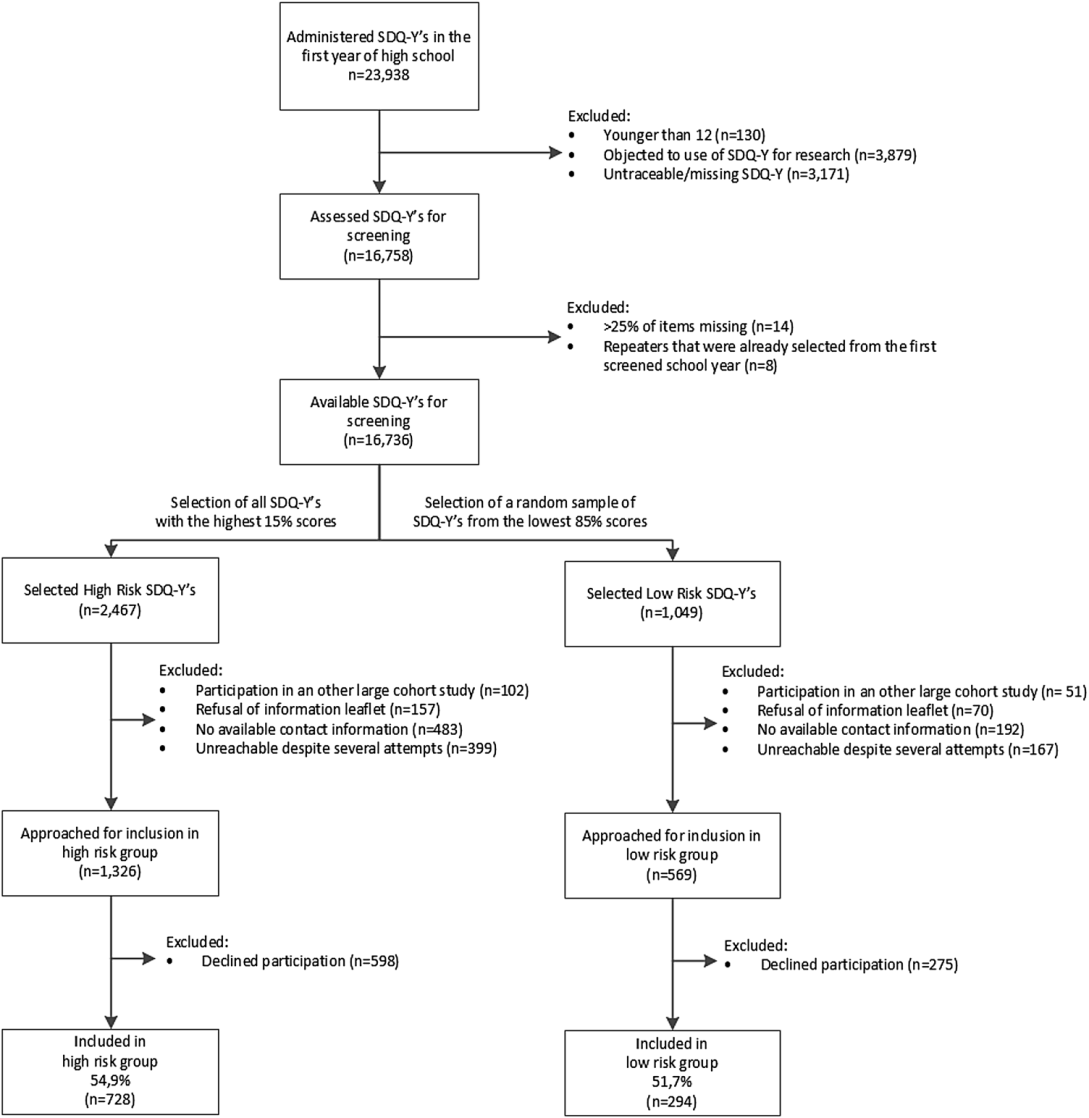
Flowchart depicting the screening and recruitment of adolescents from the general population for participation in the iBerry Study

A total of 23,938 SDQ-Y questionnaires were administered in the 2014–2015 and 2015–2016 academic years. Figure 1 summarizes the screening procedure ranging from the administration of the SDQ-Y as part of the students’ routine medical examination to the participants’ inclusion in the iBerry study. After excluding adolescents who objected to participating in the study, untraceable questionnaires, questionnaires filled out by students under 12 years of age, and questionnaires in which > 25% of items were missing, we screened a total of 16,736 of the 23,938 (69.9%) completed questionnaires.

The parents of 3,516 selected adolescents (including the highest-scoring 2467 students and a random selection of 1049 students in the lowest 85th percentile) were then randomly contacted by phone by the Child and Family Center for their permission to share their contact details with the researchers for the purposes of sending an information leaflet and a verbal request to participate in the iBerry Study. Among these 3516 adolescents, 675 could not be contacted by the Child and Family Center due to missing contact information. In addition, the majority of parents/adolescents who declined to participate cited a lack of interest (55.5%) or insufficient time to participate (13.7%). Some parents declined because they considered participation too stressful for the child (10.6%) or because the family was already receiving healthcare (4.4%). We also excluded adolescents who were already participating in another large cohort study of the Erasmus University Medical Center (4.4%). Finally, after retrieving contact information from the civil registration and despite several attempts by phone, e-mail, and regular mail, 566 adolescents (16.1%) remained unreachable.

After receiving the information leaflet, a total of 1895 (1326 high-risk and 569 low-risk) adolescents were randomly contacted by the researchers and invited to participate, with 873 adolescents declining to participate. The most commonly cited reasons for declining to participate were a lack of interest (53.8%), insufficient time to participate (18.5%), and the parent’s belief that participating would be too stressful for the child (12.2%). The final cohort consisted of 1022 adolescents, with 728 adolescents in the high-risk group and 294 adolescents in the low-risk group.

To investigate possible selection effects, we first compared the SDQ-Y scores and characteristics at the group level between the adolescents who gave passive consent for screening for research purposes and the total group of adolescents assessed during their routine preventive healthcare examination (Table 1). Because the CJG Rijnmond provided anonymous data for all administered SDQ-Ys at the group level, the scores and characteristics could not be compared using statistical analyses. Nevertheless, we found no indication of differences between the administered and screened questionnaires with respect to the adolescents’ age, gender, education level, or SDQ-Y scores. In contrast, we found subtle differences in urbanization, a higher percentage of the screened adolescents lived in the greater Rotterdam area compared to the total group of adolescents.

**Table 1:** Emotional and behavioral problem scores and socio-demographic characteristics of all assessed adolescents in general preventive healthcare and the adolescents who were screened for this study

	All assessed adolescents as part of routine youth healthcare	Adolescents screened for research purposes
	n = 23,938^a^	n = 16,736^b^
Age, years (mean ± SD)	13.1 ± 0.53	13.1 ± 0.50
Gender		
Male	12,081 (50.6%)	8465 (50.6%)
Female	11,816 (49.4%)	8271 (49.4%)
Education level		
Special secondary education	301 (2.0%)	329 (2.0%)
Pre-vocational secondary education	12,732 (53.5%)	8768 (52.4%)
Senior general secondary education	7040 (29.6%)	5137 (30.7%)
Pre-university education	3715 (15.6%)	2491 (14.9%)
Urbanization		
Rotterdam	9328 (39.0%)	5698 (34.1%)
Greater Rotterdam area	7212 (30.2%)	6345 (37.9%)
Province	7354 (30.8%)	4690 (28.0%)
SDQ-Y total score, median (range)	8 (0–31)	8 (0–33)

^a^Age, gender, education level, and urbanization information was missing for 41, 41, 150, and 44 adolescents, respectively.

^b^Age, education level, and urbanization information was missing for 669, 11, and 3 adolescents, respectively.

Table 2 compares the SDQ-Y-scores and socio-demographic characteristics between the adolescents included in the cohort and the adolescents who declined to participate, showing no significant difference with respect to gender. On average, the participating adolescents were in a higher high school level compared to the non-participating adolescents. Moreover, the percentage of high-risk adolescents living outside of the city of Rotterdam was higher among the participating adolescents than among the non-participating adolescents. Finally, participating low-risk adolescents reported more hyperactivity/inattention problems than non-participating adolescents.

**Table 2:** Socio-demographic characteristics and emotional and behavioral problem scores at time of the screening of the adolescents approached for participation to the study

	High-risk				Low-risk		
	Participated	Declined			Participated	Declined	
	(n = 728)	(n = 598)			(n = 294)	(n = 275)	
Age, years^a^ (M, SD)	13.1 ± 0.50	13.2 ± 0.49	t (1269) = 2.985, p = 0.003		13.1 ± 0.48	13.1 ± 0.49	t (558) = -0.606, p = 0.545
Gender			χ2 (1) = 0.492, p = 0.483				χ2 (1) = 0.082, p = 0.774
Male	356 (48.9%)	304 (50.8%)			144 (49.0%)	138 (50.2%)	
Female	372 (51.1%)	294 (49.2%)			150 (51.0%)	137 (49.8%)	
Education level			χ2 (3) = 16.150, p = 0.001				χ2 (3) = 13.586, p = 0.004
Special needs secondary education	14 (1.9%)	14 (2.3%)			3 (1.0%)	3 (1.1%)	
Pre-vocational secondary education	400 (54.9%)	387 (64.7%)			117 (39.8%)	151 (54.9%)	
Higher general secondary education	227 (31.2%)	153 (25.6%)			106 (36.1%)	78 (28.4%)	
Pre-university education	87 (12.0%)	44 (7.4%)			68 (23.1%)	43 (15.6%)	
Urbanization			χ2 (2) = 7.152, p = 0.028				χ2 (2) = 1.926, p = 0.382
Rotterdam	176 (24.2%)	180 (30.1%)			66 (22.4%)	75 (27.3%)	
Greater Rotterdam area	302 (41.5%)	244 (40.8%)			124 (42.2%)	105 (38.2%)	
Province	250 (34.3%)	174 (29.1%)			104 (35.4%)	95 (34.5%)	
Emotional and behavioral problems score (SDQ-Y median and range)							
Total	17 (14–30)	17 (14–32)	U = 213,579.5, p = 0.552, r = -0.016		8 (0–14)	6 (0–14)	U = 31,671.5, p < 0.001, r = -0.189
Emotional symptoms	5 (0–10)	5 (0–10)	U = 211,364.0, p = 0.359, r = -0.025		1 (0–8)	1 (0–8)	U = 36,747.5, p = 0.055, r = -0.081
Conduct problems	3 (0–9)	3 (0–9)	U = 207,401.5, p = 0.132, r = -0.041		1 (0–5)	1 (0–6)	U = 37,903, p = 0.178, r = -0.056
Hyperactivity/inattention	7 (0–10)	7 (1–10)	U = 207,201.5, p = 0.127, r = -0.042		4 (0–9)	2 (0–8)	U = 30,453, p < 0.001, r = -0.215
Peer problems	3 (0–9)	3 (0–9)	U = 213,503.0, p = 0.542, r = -0.017		1 (0–6)	1 (0–6)	U = 39,882.5, p = 0.770, r = -0.012

^a^Age was missing for 55 high-risk adolescents and 9 low-risk adolescents

### Baseline assessment procedure

The baseline characteristics of the participating adolescents were assessed between September 2015 and September 2019. During their visit to the research center, each adolescent and either one or both parents were assessed using a series of questionnaires, interviews, cognitive tests, and biological measures. After this visit, additional questionnaires were sent to one of each adolescent’s teachers and to the second parent if they did not accompany the adolescent to the research center. Both 9 and 18 months after the initial visit, a short additional questionnaire was sent to the adolescents. Additional information regarding the adolescent’s health and development can be requested via the electronic health records from the general practitioner, medical specialist, and/or other healthcare provider. During the baseline assessment researchers were blinded from the adolescents SDQ-Y score and risk status.

## Measurements

### Main outcomes

The development of psychopathology is the primary outcome in the iBerry Study. Because childhood psychiatric disorders are conceptualized as informant-specific phenomena, information obtained from multiple informants is important in order to accurately chart mental health [24]. Therefore, information was obtained from the adolescents, their parent(s), a teacher, and a clinician. Trained clinicians interviewed each adolescent using the Mini Neuropsychiatric Interview for Children and Adolescents (MINI-KID), a semi-structured diagnostic interview used to classify 26 of the most common diagnoses based on the Diagnostic and Statistical Manual IV-TR (DSM IV-TR), which was still being used by Dutch clinicians at the start of the iBerry Study. The MINI-KID interview covers attention-deficit and disruptive behavior, tics, and substance-related, psychotic, mood, anxiety, eating, and adjustment disorders [25].

To measure emotional and behavioral problems we used the Achenbach System of Empirically Based Assessment (ASEBA) questionnaires, which contain the following eight subscales: Anxious/Depressed, Withdrawn/Depressed, Somatic Complaints, Social Problems, Thought Problems, Attention Problems, Rule-Breaking Behavior, and Aggressive Behavior. These eight subscales can be combined into Internalizing Problems, Externalizing Problems, and Total Problem scales. Moreover, the following six DSM-5 oriented problem scales can be derived: Depressive, Anxiety, Somatic, Attention-deficit/hyperactivity, Oppositional defiant, and Conduct problems [26].

Supplementary Table S1 provides a complete overview of all measurements obtained during the baseline assessment. Secondary outcomes include a comprehensive assessment of substance abuse, psychotic symptoms, suicidality, self-injury, addiction to social media and/or video gaming, delinquency, and psychopathy. Additional secondary outcomes include social development and the utilization of healthcare services and social services.

### Main determinants

The main determinants in this study include the parent’s psychopathology as well as life events, contact with peers, impulsivity, lifestyle, cognitive control, somatic complaints, family functioning, and neuropsychological functioning (see Supplemental Table S1), many of which were assessed in both the adolescent and the accompanying parent(s).

### Biological samples

Biological measures included puberty development, Tanner stage (also known as the Sexual Maturity Rating), body mass index (BMI), hair samples to measure the hormone profile (including cortisol and testosterone), and blood samples for measuring genetic variants. With the exception of the puberty measurements, all measures and samples were obtained from both the adolescent and the accompanying parent(s).

## Objectives

The iBerry Study is designed to investigate the etiology of mental health problems, in particular how mental vulnerability in adolescence can lead to psychiatric disorders in adulthood.

The cross-diagnostic design of this study facilitates the study of transdiagnostic stages and trajectories of various psychiatric disorders. We expect that subclinical symptoms in adolescence have an undifferentiated pattern that shares common genetic, environmental, and pathophysiological causes.

Specifically, we will study which demographic factors (e.g., socio-economic status, cultural background), intrapersonal factors (e.g., cognition, temperament, self-esteem), interpersonal factors (e.g., peers, relationships, bullying, social media use), exogenous factors (e.g., life events, trauma), and biological factors (e.g., hormonal changes, physical health, genetic markers) underlie the transition from subclinical symptoms to psychopathology.

In addition, intergeneration factors associated with the development of psychopathology are particularly relevant to the study. We will therefore study the putative effects of parental psychopathology, parenting, and/or family functioning. Part of this intergenerational research will focus on identifying genetic determinants linked to the development of psychopathology in adolescents.

## Characteristics of the study cohort

### Socio-demographic characteristics

The socio-demographic characteristics of the participating adolescents and their parents are summarized in Tables 3 and 4, respectively.

**Table 3:** Socio-demographic characteristics of the participating adolescents at enrollment

	Total (n = 1,022)	High-risk (n = 728)	Low-risk (n = 294)
Gender			
Male	500 (48.9%)	356 (48.9%)	144 (49.0%)
Female	522 (51.1%)	372 (51.1%)	150 (51.0%)
Age, years (mean, SD)	15.0 ± 0.93	15.0 ± 0.96	15.0 ± 0.86
Ethnic background^a^			
Dutch	709 (77.5%)	504 (77.4%)	205 (77.7%)
Other Western	55 (6.0%)	35 (5.4%)	20 (7.5%)
Asian	30 (3.3%)	19 (2.9%)	11 (4.1%)
Surinamese	49 (5.4%)	32 (4.9%)	17 (6.4%)
Moroccan	12 (1.3%)	11 (1.7%)	1 (0.4%)
Turkish	12 (1.3%)	10 (1.5%)	2 (0.8%)
Dutch Antilles	19 (2.1%)	15 (2.3%)	4 (1.5%)
Cape Verdean	16 (1.7%)	14 (2.2%)	2 (0.8%)
Other Non-Western	13 (1.4%)	11 (1.7%)	2 (0.8%)
Education level^a^			
Special needs secondary education	36 (3.7%)	29 (4.3%)	7 (2.5%)
Pre-vocational secondary education	431 (44.9%)	339 (49.9%)	92 (32.8%)
Higher general secondary education	219 (22.8%)	147 (21.7%)	72 (25.6%)
Pre-university education	186 (19.4%)	98 (14.4%)	88 (31.3%)
Combined education level	88 (9.2%)	66 (9.7%)	22 (7.8%)

^a^Ethnic background and education level was missing for 107 and 62 adolescents, respectively

**Table 4:** Socio-demographic characteristics of the participating biological parents at enrollment

	High-risk		Low-risk	
	Mothers	Fathers	Mothers	Fathers
	(n = 614)	(n = 408)	(n = 249)	(n = 183)
Age, years (mean, SD)	45.8 ± 4.94	48.7 ± 5.16	46.2 ± 4.82	49.2 ± 5.47
Ethnic background				
Dutch	489 (75.2%)	493 (76.1%)	197 (74.6%)	217 (82.2%)
Other Western	39 (6.0%)	33 (5.1%)	26 (9.8%)	11 (4.1%)
Asian	26 (4.0%)	29 (4.5%)	13 (4.9%)	12 (4.5%)
Surinamese	35 (5.4%)	29 (4.5%)	15 (5.7%)	13 (4.9%)
Moroccan	12 (1.8%)	10 (1.5%)	1 (0.4%)	2 (0.8%)
Turkish	13 (2.0%)	15 (2.3%)	2 (0.8%)	2 (0.8%)
Dutch Antilles	7 (1.1%)	16 (2.5%)	3 (1.1%)	2 (0.8%)
Cape Verdean	14 (2.2%)	14 (2.2%)	2 (0.8%)	1 (0.4%)
Other Non-Western	14 (2.2%)	6 (0.9%)	5 (1.9%)	4 (1.5%)
Unknown	1 (0.1%)	3 (0.4%)	-	-
Net household income^a^				
≤ € 1599	88 (15.0%)	18 (4.6%)	19 (8.0%)	6 (3.5%)
€ 1600–2399	100 (17.1%)	49 (12.6%)	29 (12.2%)	18 (10.3%)
€ 2400–4399	286 (48.8%)	201 (51.5%)	125 (52.5%)	80 (46.0%)
≥ € 4400	112 (19.1%)	122 (31.3%)	65 (27.3%)	70 (40.2%)
Educational level				
Low	12 (1.9%)	14 (3.4%)	6 (2.4%)	2 (1.1%)
Intermediate	370 (60.3%)	212 (51.9%)	123 (49.4%)	86 (47.0%)
High	119 (19.4%)	88 (21.6%)	68 (27.3%)	51 (27.9%)
University	61 (9.9%)	68 (16.7%)	41 (16.5%)	37 (20.2%)
Combined educational level	52 (8.5%)	26 (6.4%)	11 (4.4%)	7 (3.8%)

^a^Net household income was missing for 39 mothers and 27 fathers; other missing information did not exceed 5 parents

The adolescent’s ethnicity was based on the parents’ country of birth and was used as an indication of the adolescent’s cultural and geographic background [27]. If the adolescent was born outside of the Netherlands, their birth country was used to define their ethnic background. Western descent was defined as being born in Europe, North America, or Oceania. Where possible, parental ethnic background was obtained from both parents; if only one parent participated in the study, information regarding the other parent’s ethnic background was obtained from the participating parent.

In the Dutch high school system, education levels are often combined in the first year and determined in the second or third year. Therefore, in Table 3 education level is presented in five categories, including a category for adolescents who started high school in a mixed level.

The majority of the parents who accompanied the adolescents to the research center were mothers (83.3%). Among the participating adolescents, 74.5% lived with both parents, 15.1% lived exclusively with one biological parent, 7.7% alternated between their biological parents, and 2.7% lived with adoptive parents, foster parents, or grandparents. The information provided in Table 4 was based solely on biological parents.

### Emotional and behavioral problems

Table 5 summarizes the emotional and behavioral problems reported by the participating adolescents. Emotional and behavioral problems were measured using the ASEBA questionnaires, which were completed for each adolescent by the adolescent him/herself (self-reported), both parents, and/or a teacher. A total of four, three, two, and one questionnaires was available for 36.1%, 33.7%, 20.8%, and 7.6% of adolescents, respectively; no questionnaires were completed for the remaining 1.8% of adolescents.

**Table 5:** Emotional and behavioral problems of the participating adolescents at enrollment

	High-risk (n = 712)		Low-risk (n = 292)	
	Median (range)	Percentage above borderline cut-off	Median (range)	Percentage above borderline cut-off
Self-report by the adolescent^a^				
Internalizing problems	12 (0–55)	32.5%	8 (0–40)	12.0%
Externalizing problems	10 (0–41)	19.4%	6 (0–29)	3.5%
Total problems	45 (2–141)	30.7%	30 (0–90)	8.1%
Reported by the parent that accompanied the adolescent^b^				
Internalizing problems	8 (0–51)	35.9%	5 (0–27)	14.0%
Externalizing problems	6 (0–39)	21.0%	2 (0–30)	4.7%
Total problems	30 (1–125)	35.1%	14 (0–76)	10.2%
Multi-informant (adolescent, parents(s), teacher)^c^				
% one or more informant above borderline cut-off				
Internalizing problems		55.2%		29.1%
Externalizing problems		37.9%		12.3%
Total problems		54.8%		21.6%

^a^Self-report was available in 97%

^b^A parent reported in 90% of cases

^c^A second parent reported in 59% of cases, and a teacher reported in 55% of cases

With respect to internalizing problems, 55.2% of high-risk adolescents and 29.1% of low-risk adolescents scored in the borderline/clinical range. With respect to externalizing problems, 37.9% of high-risk adolescents and 12.3% of low-risk adolescents scored in the borderline/clinical range. More than half (54.8%) of all high-risk adolescents had a total problem score in the borderline/clinical range, compared to 21.6% of low-risk adolescents.

### Adolescent psychopathology

The participating adolescents were also interviewed in order to determine the presence of DSM-IV diagnoses; these results are presented in Table 6. The most common diagnosis among the adolescents was anxiety (23.5%), followed by mood (21.1%), attention-deficit hyperactivity (ADHD,19.0%), and disruptive behavior (12.1%) disorders. Overall, the prevalence of DSM-IV diagnoses was higher in the high-risk group than in the low-risk group.

**Table 6:** Adolescent psychopathology assessed using a structured clinical DSM-IV interview at enrollment

	Total (n = 969)	High-risk (n = 688)	Low-risk (n = 281)
Mood disorders	204 (21.1%)	176 (25.6%)	28 (10.0%)
Anxiety disorders	228 (23.5%)	192 (27.9%)	36 (12.8%)
Substance-related disorders	69 (7.1%)	56 (8.1%)	13 (4.6%)
ADHD	184 (19.0%)	171 (24.9%)	13 (4.6%)
Disruptive behavior disorders	117 (12.1%)	104 (15.1%)	13 (4.6%)
Tic disorders	11 (1.1%)	9 (1.3%)	2 (0.7%)
Psychotic disorders	27 (2.8%)	25 (3.6%)	2 (0.7%)
Eating disorders	10 (1.0%)	9 (1.3%)	1 (0.4%)
Adjustment disorders	11 (1.1%)	9 (1.3%)	2 (0.7%)
No psychopathology	361 (37.3%)	192 (27.9%)	169 (60.1%)
One diagnosis	257 (26.5%)	194 (28.2%)	63 (22.4%)
Multiple diagnoses	351 (36.2%)	302 (43.9%)	49 (17.4%)

### Parental psychopathology

Table 7 summarizes the lifetime prevalence of psychopathology among the parent(s) who accompanied the adolescent to the baseline assessment. The most commonly reported disorders were mood (31.2%) and anxiety (28.9%) disorders. Moreover, 55.5% of parents met the criteria for one or more DSM-IV diagnoses within their lifetime; this rate is higher than expected, given that the adult lifetime prevalence of having any DSM-IV diagnosis has been estimated at approximately 43% in the Dutch general population [2].

**Table 7:** Lifetime parental psychopathology assessed using a structured clinical DSM-IV interview at enrollment

	Total (n = 913)	High-risk (n = 649)	Low-risk (n = 264)
Mood disorders	285 (31.2%)	212 (32.7%)	73 (27.7%)
Anxiety disorders	264 (28.9%)	194 (29.9%)	70 (26.5%)
Substance-related disorders	105 (11.5%)	81 (12.5%)	24 (9.1%)
ADHD and disruptive behavior disorders	32 (3.5%)	24 (3.7%)	8 (3.0%)
Somatoform disorders	98 (10.7%)	78 (12.0%)	20 (7.6%)
Eating disorders	33 (3.6%)	28 (4.3%)	5 (1.9%)
Psychotic disorders	20 (2.2%)	16 (2.5%)	4 (1.5%)
Adjustment disorders	32 (3.5%)	23 (3.5%)	9 (3.4%)
No history of psychopathology	407 (44.6%)	270 (41.6%)	137 (51.9%)
One diagnosis	237 (26.0%)	177 (27.3%)	60 (22.7%)
Multiple diagnoses	269 (29.5%)	202 (31.1%)	67 (25.4%)

### Statistical power

To determine the effect sizes that can be detected, we used an alpha value of 0.05 and 80% power. Depending on the prevalence of a dichotomous exposure, the study has the power to detect a difference in standard deviation ranging from 0.18 (50% prevalence) to 0.41 (5% prevalence). We consider these power calculations to be conservative, given that we will study the effect of continuous determinants and prognostic factors assessed at multiple time points during this longitudinal study.

### Data quality, control, and management

All measurements will be collected using standard protocols, and all researchers involved in the iBerry Study are fully trained and are up-to-date regarding these protocols. Quality checks of the data will be performed at regular intervals to identify inconsistencies, and any changes to the data will be logged electronically.

### Privacy protection

The iBerry Study is fully compliant with all national and European laws and regulations, including the General Data Protection Regulation and the Good Clinical Practice guideline. To ensure confidentiality of the data, all collected data will be recorded using a unique identification number for each participant. Before the data are distributed to researchers, this unique identification number and any other potentially identifying information will be excluded from the dataset, creating a fully anonymized dataset. All data will be stored on and accessed from secure servers at Erasmus University Medical Center.

### Follow-up and retention strategies

If participants were unable to make their initial appointment, it was possible to delay the appointment for up to 3–6 months or to perform the measurements during a home visit. As an incentive, the adolescent received a gift certificate and any money they won in the gambling task, as well as a booklet containing some of their test results. Where applicable, travel expenses were reimbursed. Participating families who completed the questionnaires were also entered into a lottery, with family trips as prizes. To stay in contact with the participants, we use social media channels and send birthday and holiday cards, as well as a newsletter sent at regular intervals. All contact information is verified for accuracy at each contact moment.

## Strengths and limitations

One of the main strengths of this study is its prospective, cross-diagnostic design for studying the development of psychopathology. Moreover, high-risk adolescents were selected from the general population based on their self-reported emotional and/or behavioral problems. The efficacy of the oversampling procedure based on self-reported problems is supported by our results, as apparent clinical/subclinical symptoms in the high-risk adolescents and an increased prevalence of both adolescent psychopathology and parental psychopathology were observed in the cohort compared to the general population [2, 3, 28, 29]. Thus, a higher percentage of subjects will likely be affected by traits of interest in this study. The cross-diagnostic design will enable us to study the transition from non-specific symptomatology in adolescence to the development of a full-blown disorder later in life. Another strength is that our measurements focus on a broad spectrum of prognostic factors and determinants of various types of psychopathology. Furthermore, we measure psychopathology using a multi-informant, multi-method approach, which may provide a better assessment of adolescent behavior in various contexts.

Despite these strengths, our study has several limitations that warrant discussion. The main limitation of this study is possible selection bias. However, our response rate of 53.9% is similar to the response rates reported for studies that collected data from adolescents in the same age group or in clinical/subclinical populations (25–50%) [30–35]. Although we observed no indications of attrition effects between all adolescents assessed as part of the general preventive healthcare system and the adolescents that we screened for our study, we cannot rule out the possibility of selective attrition, as not all selected adolescents participated in the cohort. Interestingly, these selection effects do not necessarily indicate that the adolescents assessed at baseline represent a selection of healthier participants [36].We observed, if anything, a slightly higher response rate among high-risk group adolescents (54.9%) compared to low-risk adolescents (51.7%). This finding suggests a possible selective non-response in adolescents who do not experience problems and is consistent with other studies suggesting that individuals who consider themselves to be low-risk are more likely to decline to participate, while individuals with a personal interest are more likely to participate [37–39]. This possible selection bias may limit statistical inference to the source population but not necessarily the scientific inference of our findings. As our study is focused on the association between variables of interest, obtaining a truly representative sample is not necessarily required [40].

Using the SDQ-Y to select adolescents may also represent a potential limitation. Adolescents were selected based solely on their self-reported emotional and behavioral problems, whereas multi-informant measures are considered the golden standard in child and adolescent psychiatry [41]. Every informant may contribute unique information, but adolescents are considered essential for reporting their symptoms, given that parents and teachers may be less aware of any problems that the adolescent may be experiencing [42, 43]. Moreover, although SDQ-Y scores can vary over time, we used only one time point to select participants. However, despite the delay between this single measurement and the baseline visit, a substantial percentage of participants showed significant problems at baseline, and extensive repeated measurements at follow-up visits will be used to study the transition from symptoms to disorders.

## Collaboration

Other researchers are welcome to collaborate with researchers in the iBerry Study group and to request access to the data. Proposals to collaborate will be assessed by the iBerry Study group with respect to quality, feasibility, and potential overlap with planned or published publications. Research proposals must be approved by the Medical Ethics Research Committee of Erasmus University Medical Center.

## Future perspectives

Currently, participants are being invited for their first follow-up visit. The use of longitudinal data collected with repeated assessments using the same assessment instruments will enable us to study the transition from subclinical symptoms to psychiatric disorders. Our goal is to follow the adolescents in our study into adulthood over a ten-year period. Investigating the prognostic, transdiagnostic, and intergenerational factors in the high-risk cohort will provide the unique opportunity to determine the individual, combined, and additive effects of these factors to the onset of psychopathology.

## Supplementary Information

Below is the link to the electronic supplementary material.

Supplementary file1 (DOCX 37 KB)
