# Supplementary material for: The iBerry study: a longitudinal cohort study of adolescents at high risk of psychopathology
Source: Eur J Epidemiol. 2021 Apr 1;36(4):453–64. doi: 10.1007/s10654-021-00740-w (PMC8076148; doi:10.1007/s10654-021-00740-w)
Supplement: Supplementary file 1 — Supplementary file1 (DOCX 37 KB) [file 10654_2021_740_MOESM1_ESM.docx]

### Supplemental Table S1. Overview of the baseline assessments.

| ***Assessment of the adolescent*** | **Instrument** | **Abbreviation** | **Type** | **Informant** |
| --- | --- | --- | --- | --- |
| General characteristics | Demographic characteristics |  | Questionnaire | Adolescent, both parents |
|  | Pregnancy, development during childhood, current health |  | Questionnaire | Parent |
| General functioning | Child Outcome Rating Scale | CORS | Self-report | Adolescent, parent, researcher |
|  | Children's Global Assessment Scale | CGAF | Assessment | Researcher |
|  | Brief Psychiatric Rating Scale for Children | BPRS-C | Assessment | Researcher |
|  | Pediatric Quality of Life Scale | PedsQL | Questionnaire | Adolescent |
| Psychopathology | Youth Self Report | YSR | Questionnaire | Adolescent |
|  | Child Behavior Checklist 6-18 | CBCL | Questionnaire | Both parents |
|  | Teacher Report Form | TRF | Questionnaire | Teacher |
| Psychiatric disorders | Mini Neuropsychiatric Interview for Children and Adolescents | MINI-KID | Interview | Adolescent |
| Psychotic symptoms | Prodromal Questionnaire 16 | PQ-16 | Questionnaire | Adolescent |
| Suicidality and self-harm | Inventory of Statements about Self-Injury | ISAS | Questionnaire | Adolescent |
|  | Questionnaire about suicidality ad self-injury | VOZZ-SCREEN | Questionnaire | Adolescent |
| Anti-social/Delinquent behavior | Self-Reported Early Delinquency Scale | SRED | Interview | Adolescent |
| Lifestyle and addiction | Substance use |  | Questionnaire | Adolescent |
|  | Social media addiction test | SMAT | Questionnaire | Adolescent |
|  | Videogame addiction test | VAT | Questionnaire | Adolescent |
| Psychopathy | Youth Psychopathic traits Inventory- Short Child Version | YPI-SCV | Questionnaire | Adolescent |
| Temperament | Early Adolescent Temperament Questionnaire – Revised | EATQ-R | Questionnaire | Adolescent |
| Health care use and costs | Trimbos/iMTA questionnaire on Costs associated with Psychiatric illness | TiC-P | Interview | Parent |
| Self | Sense of Coherence | SOC-13 | Questionnaire | Adolescent |
|  | Rosenberg Self Esteem Scale | RSES | Questionnaire | Adolescent |
| Family functioning | Family Assessment Device | FAD-12 | Questionnaire | Parent |
| Expressed emotions | Five Minute Speech Sample | FMSS | Interview | Adolescent, parent |
| Parenting | Conflict Tactics Scale Parent-Child | CTSPC | Questionnaire | Adolescent, parent |
|  | Parent-child interaction questionnaire | OKIV | Questionnaire | Adolescent, parent |
| Peers | Bullying questionnaire |  | Questionnaire | Adolescent |
| Relationships | Multidimensional Scale of Perceived Social Support | MSPSS | Questionnaire | Adolescent |
| Life events | Major life events interview |  | Interview | Parent |
| Neuropsychological functioning | IQ - Snijders-Oomen Non-verbal Intelligence test | SON-R | Test | Adolescent |
| Risky decision making | IOWA Gambling task | IOWA GT | Test | Adolescent |
| Somatic complaints | Physical Complaints Questionnaire | LKV | Interview | Adolescent |
| Anthropometry | Waist circumference |  | Assessment | Adolescent |
| Height and weight | Body Mass Index | BMI | Assessment | Adolescent |
| Body image |  |  | Questionnaire | Adolescent |
| Puberty development | Questionnaire and Tanner stadia |  | Questionnaire | Adolescent |
| Biological samples | Blood sample |  | Sample | Adolescent |
|  | Hair sample |  | Sample | Adolescent |
| ***Assessment of the parent at baseline*** | |  |  | |
| General characteristics | Demographic characteristics, health* |  | Questionnaire | Parent |
| General functioning | Global Assessment of Functioning | GAF | Assessment | Researcher |
| Psychopathology | Mini Neuropsychiatric Interview | MINI-PLUS | Interview | Parent |
|  | Brief Symptom Inventory* | BSI | Questionnaire | Parent |
| Personality | Standard Assessment of Personality Abbreviated Scale* | SAPAS | Questionnaire | Parent |
| Substance use | * |  | Questionnaire | Parent |
| Neuropsychological functioning | Snijders-Oomen Non-verbal Intelligence test | SON-R | Test | Parent |
| Health care use and costs | Treatment inventory Cost in Psychiatric patients | TiC-P | Interview | Parent |
| Anthropometry | Waist circumference |  | Assessment | Parent |
| Height and weight | Body Mass Index | BMI | Assessment | Parent |
| Biological samples | Blood sample |  | Sample | Parent |
|  | Hair sample |  | Sample | Parent |
| * Where possible, these measurements were also assessed from the second parent or caregiver. | |  |  |  |
